# A dual granular balanced deep forest model for effective drug combination prediction

**DOI:** 10.1016/j.isci.2026.116014

**Published:** 2026-06-03

**Authors:** Zhirui Gong, Ruijiang Li, Kunhong Liu, Yong Xu, Xiaocheng Bo, Song He

**Affiliations:** 1 School of Film, Xiamen University, Xiamen, China; 2 Academy of Military Medical Sciences, Beijing, China; 3 Xiamen Key Laboratory of Intelligent Fishery, Xiamen Ocean Vocational College, Xiamen, China

**Keywords:** bioinformatics, pharmacoinformatics

## Abstract

The treatment of complex diseases often benefits from combination therapies, yet identifying synergistic drug pairs remains challenging due to the vast search space and the extreme imbalance between synergistic and non-synergistic outcomes in available data. Here, we present a deep-forest-based framework designed to improve synergy prediction under highly skewed class distributions by prioritizing informative and uncertain training examples during learning. Experiments demonstrate that our method consistently achieves favorable results relative to a broad set of representative canonical, imbalanced learning, and drug-specific prediction models. Beyond predictive accuracy, we provide model interpretability analyses to highlight chemical substructures and cell-line-specific genetic signals associated with synergy, and we further validate top-ranked predictions through literature- and database-supported case studies. Together, these results suggest a practical and interpretable approach for accelerating the discovery of biologically plausible synergistic drug combinations.

## Highlights

• DGBDF targets rare drug synergies under extreme class imbalance
• Hardness- and uncertainty-guided sampling improves minority-class detection
• Dual-granularity balancing stabilizes deep forest learning across layers
• Mechanism-aware interpretability links predictions to interpretable biological evidence

## Graphical abstract

**Figure:**
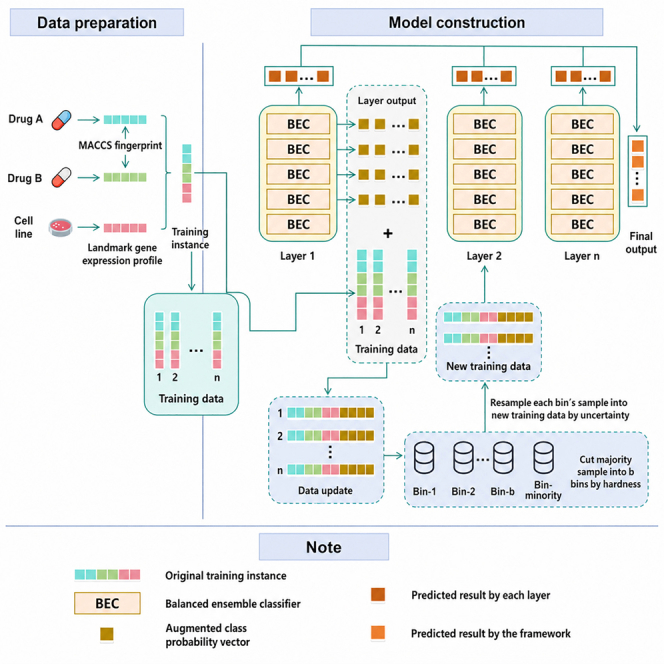

## Introduction

Drug combination therapy refers to the use of two or more therapeutic agents that interact with multiple targets simultaneously. Its clinical benefits include: (1) bypassing certain resistance mechanisms; (2) being more effective than the sum of the individual agents; (3) overcoming the toxicity and other side effects associated with high doses of a single drug.^1^^,^^2^^,^^3^ However, despite these significant clinical benefits, the sheer number of possible drug combinations makes exhaustive testing infeasible. Early research primarily relied on clinical trials, which can be unnecessary or even fatal for patients. Systematic *in vitro* experiments, such as unbiased high-throughput screening (HTS), help accelerate this process,^4^ but scanning millions of drug pairs quickly leads to a “combinatorial explosion.” Thus, algorithms are urgently needed to narrow down combinations to test.

Many machine learning algorithms have been developed for modeling structured representations of drugs and cell lines.^5^^,^^6^^,^^7^^,^^8^^,^^9^^,^^10^ Lin et al.^5^ innovatively proposed an enhanced cascade deep forest model (EC-DFR) on a small-scale drug combination dataset. Kuru et al.^11^ developed MatchMaker, a deep neural network predicting Loewe scores from drug structures and cell line gene expressions. Zhang et al.^6^ introduced KGE-DC, a knowledge graph-based model preserving relational structures. Sui et al.^10^ proposed a multi-view oblique random forest (MORF) for hepatotoxicity prediction, aiming to overcome the major barriers to hepatotoxicity identification in drug development.

Despite significant efforts to predict drug combinations, the issue of dataset imbalance has been overlooked. In classification datasets of drug combinations, effective drug combinations under specific cell lines are usually scarce. If the model does not consider the imbalance of the samples, it will tend to favor the negative (majority) class during actual predictions to achieve higher overall accuracy. This situation greatly reduces the model’s effectiveness because, in the real-world drug development process, the samples predicted as positive are those that will be truly focused on.

Previous methods for addressing class imbalance mainly fall into two categories. At the data level, re-sampling strategies aim to rebalance the class distribution by modifying the training data. Representative examples include SMOTE,^12^ which synthetically generates minority samples through feature-space interpolation, and NearMiss,^13^ which selectively removes majority samples based on distance criteria. While effective in certain scenarios, these methods often suffer from high computational cost on medium-to large-scale datasets and may introduce noise or distort the original data distribution, particularly in high-dimensional biomedical settings. At the algorithm level, imbalance is typically handled by modifying the learning objective, such as assigning different misclassification costs to different classes. Cost-sensitive learning methods^14^ require prior specification of cost matrices, which usually relies on domain expertise and is difficult to determine reliably for complex tasks like drug combination prediction. As a result, these approaches may lack flexibility when facing highly imbalanced and heterogeneous biological data.

To overcome these limitations, this study adopts the DF framework,^15^ a non–neural-network (non-NN) deep learning architecture composed of layered ensembles of decision-tree-based classifiers. Unlike traditional neural networks that rely on gradient-based backpropagation, DF is trained in a layer-wise manner, where each layer transforms the input features into class probability representations that are further refined by subsequent layers. This design enables flexible model complexity growth without extensive hyperparameter tuning. DF has attracted increasing attention and has been successfully applied in various domains,^16^^,^^17^ particularly in bioinformatics and biomedical data analysis.^18^^,^^19^ Its ensemble-based structure is inherently robust to feature heterogeneity, noise, and class imbalance, which are common challenges in drug combination prediction. These properties make DF a suitable backbone for modeling highly imbalanced drug synergy datasets.

**Figure 1:**
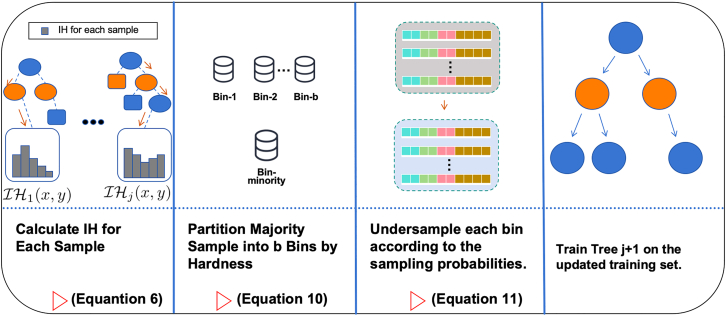
*bec* Schematic illustration of the balanced ensemble classifier (BEC) with inter-tree balancing. The steps are as follows: (1) compute $IH_{j}\left(x,y\right)$ for each sample (Equation 6); (2) partition majority samples into b hardness bins (Equation 10); (3) undersample each bin using its sampling probability (Equation 11); (4) train Tree j+1 on the updated training set. This resampling—training cycle repeats until the predefined number of trees is reached, resulting in a balanced forest for the BEC.

We propose an improved model based on DF, called dual granular balanced (DGBDF). It introduces a Balancing Module leveraging instance hardness and uncertainty, enabling informed sampling. DGBDF performs balancing at two granularities: within layers via customized balanced ensemble classifiers (BEC; Figure 1), and between layers by using prior outputs to guide sampling. Performance is measured by f1-macro after each new layer, and growth stops when improvements plateau. Finally, a cascade weighted soft voting integrates outputs across layers.

In this study, we aim to develop a DF-based imbalanced-learning framework to reliably identify synergistic drug pairs across diverse cell-line contexts under extreme class imbalance. We hypothesize that hardness- and uncertainty-guided dual-granularity balancing will emphasize boundary-defining, informative cases, improving detection of rare synergistic interactions while maintaining stringent control over non-synergistic predictions.

## Results

### Dataset and preprocessing

We derived our drug combination dataset from the screening study of Jaaks et al.^20^ In the original screen, 2025 drug combinations were evaluated on 125 cancer cell lines (breast, colorectal, and pancreatic), yielding 108259 drug-combination-cell-line measurements. In our work, each record is treated as one *drug-combination–cell-line* sample for binary synergy prediction.

#### Sample definition

Each sample can be abstractly written as:

(Equation 1) $$\left({\text{drug}}_{1},{\;\text{drug}}_{2},\text{cell},y\right)\text{.}$$

#### Drug features

For each drug, we mapped the compound identifier to a PubChem CID when available, and encoded it using the 166-bit MACCS fingerprint generated by RDKit. Let $x^{\left(1\right)}\in R^{166}$ and $x^{\left(2\right)}\in R^{166}$ denote the fingerprints of ${\text{drug}}_{1}$ and ${\text{drug}}_{2}$, respectively.

#### Cell-line features

For cell lines, we used RMA-normalized gene expression profiles from Iorio et al*.*^21^ The landmark gene list was obtained from Subramanian et al*.*^22^ We retained the expression levels of 890 landmark genes to form the cell-line feature vector $x^{\left(c\right)}\in R^{890}$.

#### Targets

We use the binary label y∈{0,1} provided in the public release of the Jaaks et al. dataset, where 1 denotes synergy and 0 denotes non-synergy.

#### Feature construction

The final input feature vector for each sample is constructed by concatenation:

(Equation 2) $$x=\left[x^{\left(1\right)};x^{\left(2\right)};x^{\left(c\right)}\right]\in R^{1222}\text{.}$$

In our implementation, $x^{\left(1\right)}$ corresponds to dimensions 1–166, $x^{\left(2\right)}$ corresponds to dimensions 167–332, and $x^{\left(c\right)}$ corresponds to dimensions 333–1222.

#### Preprocessing and filtering

We aligned drug and cell-line identifiers across data sources and removed records with incomplete feature information, including (1) drug pairs that could not be mapped to valid PubChem CIDs or MACCS fingerprints, and (2) cell lines that could not be matched to an 890-dimensional landmark-gene expression vector, (3) any record with incomplete feature values after feature construction. After filtering, we obtained 57,246 samples in total.

#### Class distribution and unique entities

Among the 57,246 samples, there are 2,183 positive (synergistic) samples and 55,063 negative (non-synergistic) samples, resulting in an imbalance ratio (IR) of approximately 25.3:1. After the above preprocessing, the dataset contains 61 unique drugs and 121 unique cell lines.

### Setup details

All experiments were performed using stratified 5-fold cross-validation, with 4-folds for training and one for testing in each round. Canonical algorithms were implemented via scikit-learn,^23^ imbalanced learning models via the imbalanced ensemble package,^24^ and existing drug combination prediction methods through publicly available code.

For our model, we tuned hyperparameters including the number of decision trees (n_estimators), scaling parameters γ and σ, the number of bins *b*, and the bin capacity parameter η in Equation 9. Optimal values, highlighted in Table 1, were selected after comparative experiments. All baseline methods were likewise carefully tuned to ensure a fair comparison, with configurations detailed in Tables S7–S9.

**Table 1:** Concise title without reference or footnote citations

Hyperparameter	Values Considered
n_estimators	10, **20**, 50
γ, δ	[0.1, 1], [0.1, 5], **[0.5, 5]**
b	3, **5**, 10
η	0.01, 0.05, **0.1**, 0.5, 1

### Comparison between different models

To validate the effectiveness of our model, we comprehensively compared it with canonical, imbalanced learning, and several existing drug combination prediction algorithms. The description of the imbalanced learning algorithms and existing drug combination prediction algorithms compared in the study is presented in Table S1.

#### Compared with canonical algorithms

**Figure 2:**
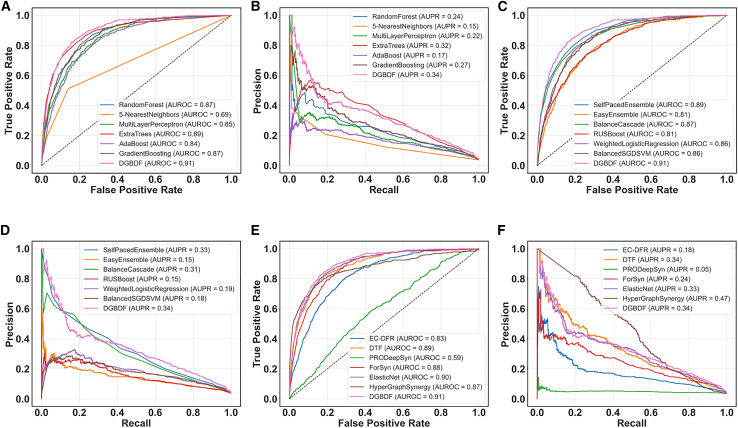
AUC_AUPR Receiver operating characteristic (ROC) and precision—recall (PR) curves comparing DGBDF with three groups of baselines. Curves were generated from pooled test-set predictions across cross-validation folds. (A and B) Canonical classifiers; (C and D) imbalanced-learning methods and linear baselines; (E and F) existing drug-combination prediction models. Subplots (A, C, and E) report ROC curves and AUROC, while (B, D, and F) report PR curves and AUPR (shown in the legends). Across all three comparison groups, DGBDF achieves the highest AUROC (0.91), and it also attains the best AUPR in the canonical and imbalanced-learning comparisons (AUPR = 0.34). Against specialized drug-combination predictors, DGBDF remains competitive in AUPR (e.g., comparable to DTF/ElasticNet) although HyperGraphSynergy yields a higher AUPR (0.47), highlighting the differing emphasis of these methods under extreme class imbalance.

First, we introduce some canonical algorithms for comparison. The experimental results are shown in the first block of Table 2, Figures 2A and 2B.

**Table 2:** Performance comparison^a^

Classifier	sen	spe	f1-macro	gmean
RandomForest	0.06	**1.00**	0.54	0.24
5-NearestNeighbors	0.06	**1.00**	0.54	0.15
MultiLayerPerceptron	0.01	**1.00**	0.50	0.06
ExtraTrees	0.12	**1.00**	0.59	0.35
AdaptiveBoosting	0.03	**1.00**	0.52	0.17
GradientBoosting	0.03	**1.00**	0.52	0.19
SelfPacedEnsemble^25^	0.62	0.92	0.65	0.77
EasyEnsemble^26^	0.66	0.77	0.52	0.71
BalanceCascade^26^	0.57	0.89	0.60	0.73
RUSBoost^27^	0.75	0.51	0.73	0.53
WeightedLogisticRegression	**0.74**	0.74	0.55	0.74
BalancedSGDSVM	0.64	0.63	0.46	0.53
EC-DFR^5^	0.00	**1.00**	0.49	0.00
DTF^28^	0.00	0.99	0.62	0.41
PRODeepSyn^29^	0.51	0.55	0.39	0.53
ForSyn^8^	0.12	0.99	0.59	0.38
ElasticNet^30^	0.19	0.99	0.60	0.37
HyperGraphSynergy^31^	0.41	0.98	**0.73**	0.64
DGBDF (ours)	0.73	0.89	0.63	**0.81**

a Bold values indicate the best performance for each metric within the corresponding comparison group.

Although several canonical models exhibit seemingly reasonable AUROC values (e.g., RandomForest: 0.87, ExtraTrees: 0.89, GradientBoosting: 0.87 in Figure 2A), their classification behavior is in fact dominated by the majority (negative) class. As reported in Table 2, all canonical baselines achieve an extreme specificity of 1.00, while their sensitivity remains near zero (sen ranges from 0.01 to 0.12), indicating that they predict almost all test samples as negative and fail to capture positive (synergistic) samples. Consequently, their gmean is very low (0.06–0.35) and AUPR is also limited (0.15–0.32 in Figure 2B), reflecting poor precision-recall trade-offs under severe imbalance.

In contrast, DGBDF substantially improves positive recognition without collapsing specificity: it achieves AUROC = 0.91 and AUPR = 0.34 (Figures 2A and 2B), together with a much higher sen = 0.73 and gmean = 0.81 (Table 2). These results confirm that canonical learners are not robust to the extreme imbalance in drug synergy prediction, whereas DGBDF effectively identifies hard minority samples.

#### Compared with imbalanced learning algorithms

Since the model has been improved to address data imbalance issues, we further compare it with representative imbalance learning methods. The experimental results are shown in the second block of Table 2, Figures 2C and 2D.

Overall, DGBDF achieves the best balance between minority recall and majority rejection. Specifically, DGBDF obtains the highest gmean of 0.81, outperforming the strongest competitors such as SelfPacedEnsemble (0.77) and BalanceCascade/RUSBoost (0.73) by noticeable margins (Table 2). This gain comes from maintaining a high sensitivity (sen = 0.73) while keeping specificity at a high level (spe = 0.89), whereas some baselines trade one for the other (e.g., WeightedLogisticRegression has comparable sen = 0.74 but much lower spe = 0.74, leading to a lower gmean = 0.74).

The curve-based metrics show consistent trends. DGBDF achieves the best AUROC (0.91 in Figure 2C), exceeding SelfPacedEnsemble (0.89) and BalanceCascade (0.87). More importantly under imbalance, DGBDF also reaches the top AUPR (0.34 in Figure 2D), slightly higher than SelfPacedEnsemble (0.33) and BalanceCascade (0.31), indicating improved precision-recall behavior for the positive class.

#### Compared with existing drug combination prediction algorithms

To more comprehensively evaluate the model’s performance, we further compared DGBDF with advanced drug combination prediction algorithms. The experimental results are shown in the third block of Table 2, Figures 2E and 2F.

Several existing models exhibit strong majority-class preference similar to canonical baselines. For example, EC-DFR yields a sensitivity of 0.00 and a specificity of 1.00, resulting in a G-mean of 0.00. In addition, DTF, ForSyn, and ElasticNet achieve very high specificity (0.99, 0.99, and 0.99, respectively) but poor sensitivity (0.17, 0.12, and 0.19, respectively), leading to limited G-mean values (0.41, 0.38, and 0.37, respectively) (Table 2). PRODeepSyn behaves differently: it does not collapse to predicting all negatives (sen = 0.51, spe = 0.55), but its overall discrimination is weak, reflected by AUROC = 0.59 and AUPR = 0.05 (Figures 2E and 2F) and a low gmean = 0.53. Notably, HyperGraphSynergy achieves the highest AUPR (0.47 in Figure 2F) and the best f1-macro (0.73 in Table 2]), indicating strong ranking/early-retrieval ability for positives. However, its sensitivity remains moderate (sen = 0.41), which limits its gmean to 0.64.

In contrast, DGBDF provides the best overall class-balanced recognition: it achieves the highest gmean (0.81) and a substantially higher sensitivity (sen = 0.73), while maintaining a high specificity (spe = 0.89) (Table 2). In terms of discrimination, DGBDF also achieves the best AUROC (0.91 in Figure 2E). Although its AUPR (0.34) is not the top among all specialized models, it delivers a markedly better sensitivity-specificity trade-off, which is critical for reliable synergy identification under extreme class imbalance.

### Ablation experiments

**Figure 3:**
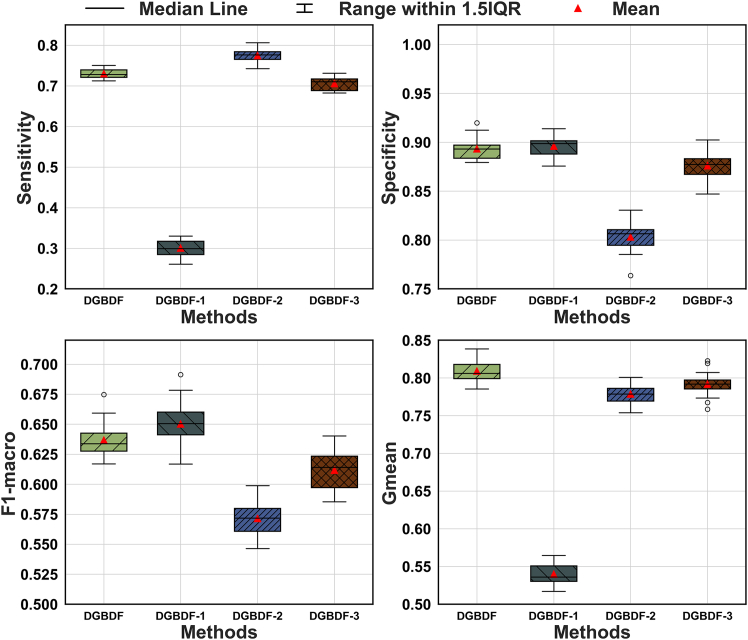
*ablation_study* Ablation study comparing the full DGBDF model with variants removing inter-tree balancing, inter-layer balancing, or cascade weighted soft voting. The center line indicates the median, the box indicates the interquartile range (IQR), whiskers indicate the range within 1.5×IQR, open circles indicate outliers, and red triangles indicate the mean. The complete model achieves the best overall performance, while removing any component leads to noticeable performance degradation, indicating that each module contributes to robust prediction under class imbalance.

To evaluate the contribution of each component in DGBDF, we conducted ablation experiments by selectively disabling inter-tree balancing, inter-layer balancing, and cascade weighted soft voting. The configurations are listed in Table 3, with results shown in Figure 3.

**Table 3:** Ablation study on different DGBDF variants

Ablation	Characteristics
DGBDF	dual granular balancing, cascade weighted soft voting
DGBDF-1	without inter-tree balancing
DGBDF-2	without inter-layer balancing
DGBDF-3	without cascade weighted soft voting

Overall, the complete DGBDF model, integrating all three modules, achieves the best performance with the highest gmean (0.81), strong sensitivity (0.73), and f1-macro (0.64). Removing any component degrades results, highlighting their necessity.

DGBDF-1, without inter-tree balancing, suffers a sharp drop in sensitivity (0.30) despite high specificity (0.98), yielding a lower gmean (0.54), and revealing a bias toward the majority class.

DGBDF-2, which omits inter-layer balancing, shows high sensitivity (0.77) but reduced specificity (0.80), resulting in the lowest f1-macro (0.57), and suboptimal gmean (0.78), underscoring the importance of balancing across layers.

DGBDF-3, which replaces cascade voting with only the final layer’s output, causes moderate declines across all metrics (f1-macro: 0.61, gmean: 0.79), demonstrating the benefit of multi-layer aggregation.

These results confirm that each component independently and collectively strengthens model robustness, while their absence notably compromises performance on imbalanced data.

### Effects of instance uncertainty measure

The introduction of uncertainty estimation serves not only to enhance model stability but more crucially to correct misclassified instances as the ensemble deepens. With the increasing number of classifiers in deeper layers, model decisions inevitably exhibit greater variance. To evaluate the effectiveness of the proposed uncertainty measure, we focus on instances exhibiting conflicting predictions—where the outputs of different classifiers for a test instance s include both probabilities <0.5 and ≥0.5. Such cases are defined as crossover situations, and typically correspond to high uncertainty scores as described by the scalar in Equation 8.

**Figure 4:**
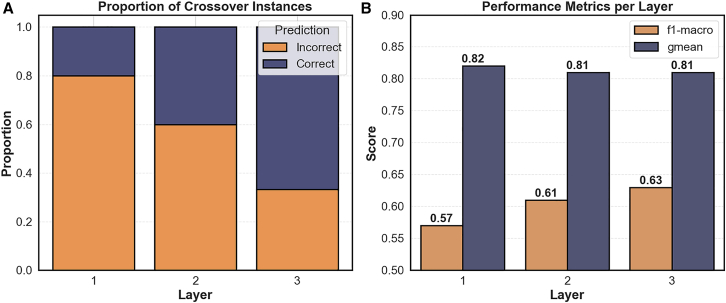
*effects_of_uncertainty* Values represent the mean across cross-validation folds. (A) We define a crossover instance as a test sample whose base classifiers within a layer produce conflicting decisions around the threshold 0.5 (i.e., some predicted probabilities are <0.5 while others are ≥ 0.5), indicating high uncertainty. Stacked bars report, for each layer output, the proportion of crossover instances that are classified correctly vs. incorrectly. The correctly predicted fraction increases with depth (layer 1: 0.20; layer 2: 0.40; layer 3: 0.67), showing that deeper layers progressively rectify ambiguous cases. (B) Overall performance per layer measured by F1-macro and G-mean. F1-macro improves consistently (0.57 → 0.61 → 0.63), while G-mean remains stable (0.82 → 0.81 → 0.81), suggesting improved class-balanced prediction (especially for minority-class detection) without sacrificing the overall balance between sensitivity and specificity.

Figure 4A presents a quantitative analysis of these crossover instances. As the number of DGBDF layers increases, the proportion of correctly predicted crossover instances steadily rises, while the incorrectly predicted ones decline. This trend demonstrates that deeper layers benefit from the uncertainty measure by focusing on high-uncertainty instances and gradually correcting errors.

In addition, we assess overall model performance across layers using F1-macro and G-mean metrics. As shown in Figure 4B, F1-macro improves consistently with depth, indicating better classification balance, especially for minority classes. Meanwhile, G-mean remains stable, suggesting that the performance on majority classes is not compromised. These results validate that the uncertainty-guided mechanism promotes reliable correction of ambiguous cases while maintaining general performance across class distributions.

### Visualizing learned representations via t-SNE

To investigate the feature representation ability of our DGBDF model, we conducted a t-SNE visualization on the drug pair-cell line embeddings. The dataset was stratified and split into training and testing sets with an 8:2 ratio, which is a commonly adopted setting that balances sufficient training data with a held-out test set for reliable qualitative evaluation. We selected three representative breast cancer cell lines—AU565, CAL-51, and BT-474—from the test set, as they exhibit stable and reliable performance for visualization.

**Figure 5:**
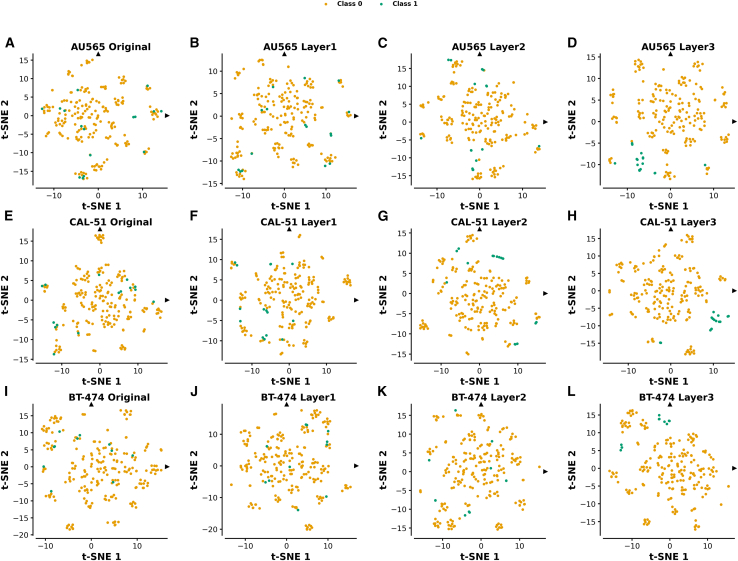
*tsne* t-SNE of layer-wise representations learned by DGBDF on three breast-cancer cell lines. Each point is a test drug-pair instance; colors denote class 0 (orange) vs.∖ class 1 (green). Panels A–D, E–H, and I–L show the original representations and representations from model layers 1–3 for AU565, CAL-51, and BT-474, respectively. Separation improves with depth, suggesting increasingly discriminative representations.

As shown in Figure 5, the “Original” column represents the raw features before entering the ensemble model, while the subsequent three columns show the augmented representations after layer 1, layer 2, and layer 3 of our model, respectively. Each point is colored according to its synergy label: class 0 (No synergy) and class 1 (Synergy). In our model, each layer not only inputs the original feature but also appends an augmented feature vector to each instance, enabling the model to incorporate additional, layer-specific discriminative information. These augmented features progressively enrich the representation space as the input flows through the ensemble layers.

From the visualization, we observe that the samples of class 1 (green) and class 0 (orange) are highly entangled in the original feature space, suggesting poor initial separability. However, as features propagate through the layers of the ensemble model—with augmentation at each stage—the distinction between the two classes becomes more pronounced, especially in layer 3. This demonstrates that the model progressively learns more discriminative information, enabling better classification of synergistic and non-synergistic drug pairs. In most cases, the cascade stops growing at layer 3 because the learned representations and predictive behavior have already stabilized; adding further layers typically yields negligible changes and does not provide clearer qualitative insights in the t-SNE plots.

### Model interpretation via SHAP-based feature importance analysis

To interpret the decision process of DGBDF in predicting synergistic drug responses, we performed SHAP (SHapley Additive exPlanations) analysis using TreeExplainer, which computes feature attributions based on Shapley values and provides efficient, exact explanations for tree ensemble models.^32^ The model takes three types of input features: MACCS structural keys for drug 1 and drug 2, and gene expression profiles of the target cell line. For interpretability, a stratified 8:2 train-test split was used to compute SHAP values and assess feature contributions.

**Figure 6:**
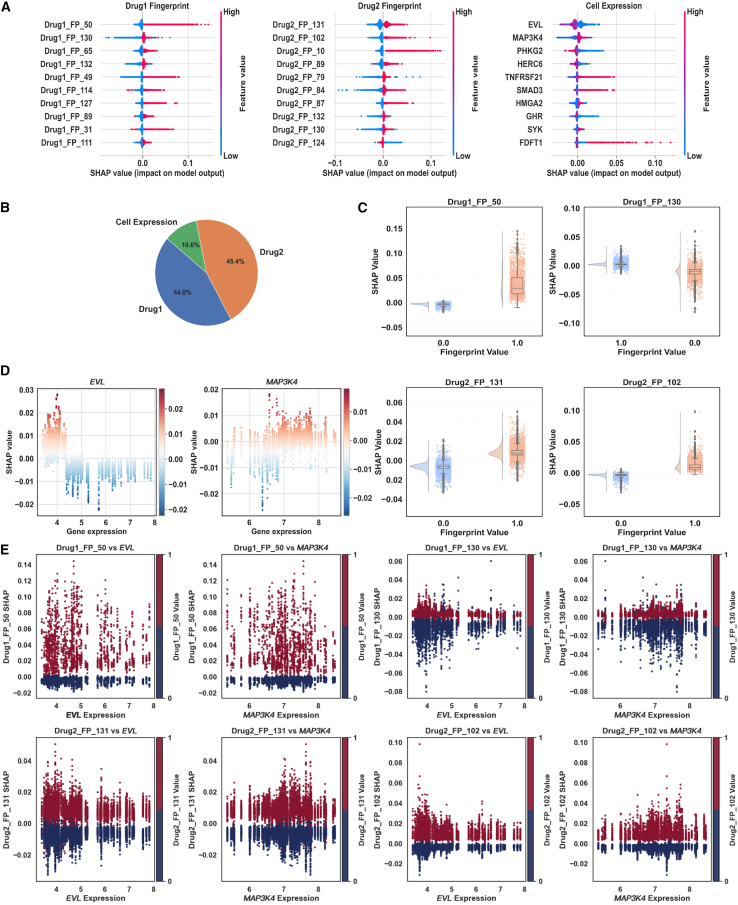
*feature_importance* SHAP-based interpretability of DGBDF using drug fingerprints and cell-line gene expression. (A–C) Global feature-attribution results: beeswarm summaries of top MACCS bits for drug 1/drug 2, group-wise contributions (mean |SHAP|) for drug 1, drug 2 and expression, and representative univariate dependence plots for high-impact fingerprint bits. (D) SHAP dependence for key genes (e.g., *EVL*, *MAP3K4*). (E) Bivariate dependence showing model-level interactions between selected fingerprint bits and gene expression.

Global feature-importance analysis identified the most influential features across drug 1 fingerprints, drug 2 fingerprints, and cell-line gene-expression profiles (Figure 6A). The top contributors included drug1_FP_50, drug2_FP_131, *EVL*, *MAP3K4*, *PHKG2*, and *HERC6*. To quantify the overall influence of each feature group, we computed mean absolute SHAP values within each modality (Figure 6B). Drug 1 and drug 2 accounted for 44.6% and 45.4% of total contributions, respectively, whereas cell-line gene-expression features contributed 10.9%, indicating that compound structure predominantly drives prediction while transcriptional context remains non-negligible.

Figure 6C shows dependence plots for drug1_FP_50, drug1_FP_130, drug2_FP_131, and drug2_FP_102, illustrating how their SHAP values vary with raw feature values. We clarify the indexing convention of the MACCS fingerprint features used during model training: we adopt 0-based feature indices, where FP-i corresponds to MACCS key (i+1). we further link representative top-ranked bits to their public key definitions. For example, drug2_FP_131 corresponds to MACCS key 132 (“OACH2A”), an oxygen atom in close topological proximity to a methylene unit. Such oxygen-containing linkage motifs are common in drug-like scaffolds and may influence physicochemical properties (e.g., polarity and hydrogen-bond acceptor capacity), thereby potentially modulating context-dependent drug responses.^33^^,^^34^ Figure 6D further examines the gene expression levels of EVL and MAP3K4, where higher expression tends to yield larger positive SHAP contributions, suggesting that these cellular-state variables systematically influence predicted synergy.

To investigate cross-modal effects between drug structure and cellular context, we analyzed bivariate SHAP dependence plots (Figure 6E). We emphasize that these plots capture model-level statistical interactions (i.e., context-dependent feature contributions learned from data) rather than direct physical interactions between a substructure and a gene product.^32^ Notably, the SHAP contribution of drug2_FP_131 is maximized at intermediate MAP3K4 expression, suggesting a “regulatory window” in which the presence/absence of the OACH2A motif is most informative for synergy prediction.

To further elucidate how DGBDF arrives at individual predictions, we analyzed local SHAP waterfall plots (Figure S2). These plots were generated for 8 randomly selected positive samples and 4 randomly selected negative samples. Each plot decomposes the prediction for a single drug-combination-cell-line instance, showing how specific features incrementally push the model output away from the baseline expectation *E*[*f*(*x*)] toward the final predicted value. In positive samples, certain drug molecular fingerprints and genes such as *EVL* and *MAP3K4* frequently contribute positive shifts, collectively driving the prediction toward higher synergy scores. Conversely, in negative samples, these or other features exert downward forces, reducing the predicted synergy likelihood. The direction and magnitude of each feature’s contribution vary across instances, highlighting the model’s sensitivity to case-specific molecular and transcriptional context.

From a biological perspective, MAP3K4 is an upstream MAPKKK that can activate the stress-responsive JNK and p38 pathways via MKKs, pathways widely implicated in cellular stress responses and apoptosis programs relevant to chemotherapeutic sensitivity.^35^^,^^36^ A plausible interpretation is that when MAP3K4-associated stress signaling is at a moderate activity state (proxied here by expression), cells may reside in a transitional susceptibility regime where drug-induced perturbations are more likely to be amplified into apoptotic outcomes; under such contexts, structural motifs that influence polarity and hydrogen-bond acceptor patterns (such as oxygen-linked fragments captured by OACH2A) may correlate with differential intracellular exposure or target engagement, thereby increasing the model-inferred synergy contribution.^33^^,^^34^ It provides a mechanistically grounded hypothesis consistent with the observed SHAP interaction pattern.

### Case study

To further substantiate the DGBDF model, we demonstrate its application across two scenarios: potential combination detection and synergistic drug prioritization.

#### Potential combination detection

Leveraging the DGBDF model, we explored potential drug combinations within the breast cancer cell line HCC1428. Out of the top 30 synergistic drug combinations predicted, at least five were confirmed through literature validation, as shown in Tables 4 and S2. Notably, the combination of navitoclax (ABT-263) and vinorelbine represents a well-characterized pathway-complementary synergistic pair. Navitoclax inhibits anti-apoptotic BCL-2 family proteins, whereas vinorelbine disrupts microtubules and induces the BH3-only protein NOXA, thereby counteracting MCL-1-mediated survival signaling. By jointly suppressing distinct anti-apoptotic branches within the intrinsic apoptotic pathway, this complementary mechanism lowers the apoptotic threshold and leads to enhanced tumor cell death.^37^ Additionally, vinorelbine enhances the sensitivity of tumor cells to venetoclax without adversely affecting normal cells. The combination of venetoclax and vinorelbine exhibited synergistic cytotoxic effects on tumor cells and demonstrated selectivity. Additional potential combinations have also been identified in various literature sources.^38^ Overall, these validated cases suggest that the majority of the predicted synergistic drug combinations act through pathway-complementary mechanisms, targeting distinct regulatory nodes within the same core biological pathways rather than converging on identical molecular targets.

**Table 4:** Top predicted synergistic drug combinations in HCC1428 and their literature validation

No	Rank	Drug1	Drug2	Evidence (PMID)	Score
1	2	Navitoclax	Vinorelbine	23723123^37^	0.81
2	3	Venetoclax	Vinorelbine	32318340^38^	0.71
3	5	AZD7762	Irinotecan	19841922^39^	0.65
4	8	Erlotinib	Venetoclax	35092513^40^	0.56
5	9	Dactolisib	Venetoclax	35568072^41^	0.55

#### Synergistic drug prioritization

In the context of synergistic drug prioritization, we utilized the DGBDF model to identify potential combinatorial drugs alongside AZD7762 in pancreatic cancer cell lines (Table S3). Our analysis revealed that the combination of AZD7762 with vorinostat (ranked 4th) and axitinib (ranked 5th) was supported by cross-validation through the DrugComb database.^42^ Mechanistically, AZD7762 influences the DNA damage response, while Vorinostat modulates gene transcription and the cell cycle, suggesting a potential synergistic effect.^43^ Axitinib, a tyrosine kinase inhibitor, primarily targets the vascular endothelial growth factor receptor (VEGFR), disrupting tumor angiogenesis and reducing nutrient supply to the tumor.^44^ Theoretically, this combination with AZD7762 could yield significant synergistic effects. The cross-validation from public databases, along with support from the literature and mechanism analysis, demonstrates the effectiveness of the prioritized drugs, further validating the utility of the model.

### Statistical comparison and significance analysis

To assess the statistical reliability of performance differences among the compared methods, we conducted a non-parametric statistical analysis based on the G-mean metric. Each method was evaluated under a 5 × 5 repeated 5-fold cross-validation protocol, yielding *N* = 25 blocks for paired statistical comparison.

The omnibus Friedman test showed a statistically significant overall difference among the compared methods (χ^2^ = 449.621, *p* = 3.897 × 10^−84^). As summarized in Table S4, DGBDF achieved the best average rank (AvgRank = 1.0), indicating consistently top-ranked performance across all repeated cross-validation blocks. Imbalance-aware baselines, including SelfPacedEnsemble and RUSBoost, ranked immediately after DGBDF, whereas canonical classifiers without dedicated imbalance-handling mechanisms, such as k-nearest neighbors and multilayer perceptron, ranked near the bottom.

To further quantify practical improvements beyond rank-based comparison, we analyzed paired block-wise G-mean differences between DGBDF and each baseline method. For each baseline, we summarized the win/tie/loss counts and reported the mean and median paired improvements with bootstrap 95% confidence intervals. As reported in Table S5, DGBDF outperformed every baseline in all 25 blocks (win/tie/loss = 25/0/0). For all baselines, the bootstrap 95% confidence intervals of both the mean and median improvements excluded zero, indicating consistently positive and statistically supported gains across repeated cross-validation splits.

Together, the Friedman ranking analysis and the paired improvement analysis support the superior and robust performance of DGBDF on this highly imbalanced drug-combination prediction task. Detailed average rankings and paired improvement statistics are provided in Tables S4 and S5.

## Discussion

Drug-combination synergy prediction is inherently difficult under severe class imbalance, where true synergies are rare and many models become majority-dominated. Our results suggest that DGBDF improves the sensitivity-specificity trade-off. Extensive experiments show that DGBDF outperforms canonical, imbalanced learning, and drug-specific baselines. In particular, DGBDF achieves sensitivity of 0.73 and specificity of 0.89, resulting in the best Gmean of 0.81, together with an AUROC of 0.91, among the compared methods in our evaluation. Ablation analyses further indicate that the observed improvements arise from the synergistic interaction between dual-granularity balancing and cascade-level integration, rather than from any individual component alone.

From a pharmacology and screening perspective, these gains are meaningful because most experimentally measured synergy signals are dose- and context-dependent, and the apparent strength of an interaction can shift with the tested concentration range and with the choice of synergy formalism. Large-scale resources (e.g., NCI-ALMANAC) demonstrated both the promise and the difficulty of translating high-throughput combination hits into actionable hypotheses, precisely because only a minority of pairs show consistent enhanced activity across conditions and because noise/heterogeneity is unavoidable in high-dimensional response surfaces.^45^^,^^46^ Related work has argued that separating “synergy of potency” from “synergy of efficacy” can change how a combination is interpreted for dose reduction vs. maximal killing, which is particularly relevant to preclinical decision-making and may explain why purely ranking-optimized models can behave differently depending on the evaluation target and operating point.^46^ In this context, DGBDF’s balanced operating characteristics are aligned with real screening workflows where investigators often prefer a model that does not disproportionately miss true synergistic candidates when synergy is rare.

Interpretability analysis reveals the complementary contributions of drug structural features and cell-line-specific gene expression profiles in determining drug synergy, consistent with the context-dependent nature of combination responses. This is consistent with prior computational synergy predictors showing that integrating chemical representations with molecular context improves predictive signal and enables hypotheses that are conditional on cellular state rather than purely drug-pair identity (e.g., deep learning synergy models leveraging chemical descriptors and transcriptomics^47^). Importantly, the observed “structure + expression” complementarity supports a biologically plausible view: chemical structure constrains target engagement and polypharmacology, whereas expression profiles proxy pathway activity and vulnerability, which together shape whether two perturbations become synergistic in a given cell line.

The case studies further support this interpretation, as many predicted combinations are corroborated by existing evidence and predominantly exhibit pathway-complementary interaction patterns. Together with its robustness under severe class imbalance, these properties make DGBDF well suited for early-stage and preclinical drug combination discovery, where synergistic effects are rare and experimental validation is costly. In practice, a model is most valuable when it can help investigators triage candidates across heterogeneous evidence sources and screening datasets; community portals that harmonize combination screens (e.g., DrugComb) were created precisely to support such reuse and prioritization.^48^ By prioritizing biologically plausible and mechanistically interpretable candidates, the proposed framework can help narrow the experimental search space, facilitate more informed study design (e.g., choosing relevant cellular contexts and follow-up assays), and support data-driven decision-making in preclinical research and precision medicine applications.

### Limitations of the study

Despite these promising results, several limitations should be acknowledged. As the study is a secondary analysis of publicly available cancer cell-line screening data, we could not assess potential sex/gender effects on drug-combination responses. Although the proposed framework effectively addresses severe class imbalance at the data and model levels, it does not explicitly incorporate higher-order biological knowledge, such as signaling network topology or pathway dynamics, which may further refine synergy interpretation. Future work will focus on extending DGBDF by integrating multi-omics data and prior biological knowledge to enhance interpretability and generalizability.

## Resource availability

### Lead contact

Requests for further information and resources should be directed to and will be fulfilled by the lead contact, Kunhong Liu (lkhqz@xmu.edu.cn).

### Materials availability

This study did not generate new materials.

### Data and code availability

#### Data

The processed datasets used in this study have been deposited in the GitHub repository and archived in Zenodo, as listed in the key resources table. They are publicly available as of the date of publication. GitHub: https://github.com/MLDMXM2017/DGBDF; Zenodo: https://doi.org/10.5281/zenodo.19814836.

#### Code

The source code and documentation for DGBDF have been deposited in the GitHub repository and archived in Zenodo, as listed in the key resources table. They are publicly available as of the date of publication. GitHub: https://github.com/MLDMXM2017/DGBDF; Zenodo: https://doi.org/10.5281/zenodo.19814836.

#### Other items

Any additional information required to reanalyze the data reported in this paper is available from the lead contact upon reasonable request.

## Acknowledgments

The authors thank the members of the research team for helpful discussions and technical support during this study. The authors also thank the editors and reviewers for their constructive comments on the manuscript. This work is supported by Joint Funds for the innovation of science and Technology, Fujian province (Grant number 2024Y9079), and the public technology service platform project of Xiamen City (No. 3502Z20231043).

## Author contributions

S.H., X.B., Y.X., and K.L. conceived the project and supervised the project. Z.G. and R.L. designed the algorithm, conducted computational experiments, wrote the manuscript, and drew the pictures. All authors approved the final manuscript.

## Declaration of interests

The authors declare no competing interests.

## STAR★Methods

### Key resources table

**Table:** 

REAGENT or RESOURCE	SOURCE	IDENTIFIER
**Deposited data**		

datasets	This paper	GitHub: https://github.com/MLDMXM2017/DGBDF; Zenodo: https://doi.org/10.5281/zenodo.19814836

**Software and algorithms**		

source code and documentation	This paper	GitHub: https://github.com/MLDMXM2017/DGBDF; Zenodo: https://doi.org/10.5281/zenodo.19814836
python	Version 3.9	https://www.python.org/downloads/release/python-390/
imbalanced-ensemble	Version 0.2.1	https://pypi.org/project/imbalanced-ensemble/0.2.1/
numpy	Version 1.24.3	https://pypi.org/project/numpy/1.24.3/
scikit-learn	Version 1.3.1	https://pypi.org/project/scikit-learn/1.3.1/

### Experimental model and study participant details

#### Cell lines

This study performed secondary analyses of publicly available drug-combination screening data derived from cancer cell lines. The dataset was obtained from the screening study of Jaaks et al.^20^ This study included pancreatic cell lines (AsPC-1, BxPC-3, CAPAN-1, CAPAN-2, CFPAC-1, DAN-G, HPAC, Hs-766T, HuP-T3, HuP-T4, KP-1N, KP-2, KP-3, KP-4, MIA-PaCa-2, MZ1-PC, PA-TU-8902, PA-TU-8988T, PANC-02-03, PANC-03-27, PANC-04-03, PANC-08-13, PANC-10-05, PL4, PSN1, SU8686, SUIT-2, SW1990, YAPC), breast cell lines (AU565, BT-20, BT-474, BT-483, BT-549, CAL-120, CAL-148, CAL-51, CAL-85-1, CAMA-1, COLO-824, DU-4475, EFM-19, EFM-192A, EVSA-T, HCC1143, HCC1187, HCC1395, HCC1419, HCC1428, HCC1500, HCC1569, HCC1599, HCC1806, HCC1937, HCC1954, HCC202, HCC2157, HCC2218, HCC38, HCC70, HDQ-P1, Hs-578-T, JIMT-1, MCF7, MDA-MB-157, MDA-MB-231, MDA-MB-330, MDA-MB-361, MDA-MB-415, MDA-MB-436, MDA-MB-453, MDA-MB-468, MFM-223, MRK-nu-1, OCUB-M, T47D, UACC-812, UACC-893, ZR-75-30), and colon cell lines (C2BBe1, CaR-1, CCK-81, CL-11, COLO-205, COLO-320-HSR, COLO-678, CW-2, GP5d, HCC2998, HCT-116, HCT-15, HT-115, HT-29, HT55, KM12, LoVo, LS-1034, LS-123, LS-180, LS-411N, LS-513, MDST8, NCI-H716, NCI-H747, RCM-1, RKO, SK-CO-1, SNU-1040, SNU-175, SNU-407, SNU-81, SNU-C1, SNU-C2B, SNU-C5, SW1116, SW1417, SW1463, SW48, SW620, SW837, SW948, T84).

No new cell-line experiments, cell culture, drug screening, or primary cell isolation were performed in this study. The cell lines analyzed here were not generated, cultured, authenticated, or tested for mycoplasma contamination by the authors. Information on cell-line provenance, culture conditions, authentication, and mycoplasma testing, where available, should be referred to the original screening study from which the data were obtained. Because this study used only publicly available cancer cell-line datasets and did not involve recruitment of human participants, animal experiments, plants, microbe strains, primary cell cultures, or newly generated biological samples, institutional approval and participant consent were not applicable.

### Method details

#### Problem definition

We formulate drug combination prediction as a binary classification task: given a drug pair (A, B) and a cell line C, predict whether they produce a synergistic effect. Let the training set be D={X,Y}, where $X\in R^{n\times m}$ contains n samples and m features, and $Y\in {\left\{0,1\right\}}^{n}$ are the corresponding binary labels. We define:

(Equation 3) P={⟨x,y⟩∣y=1},N={⟨x,y⟩∣y=0}

where each sample x corresponds to one triplet (A, B, C) (a drug pair tested on a specific cell line) and is represented by the concatenated feature vector defined in Equation 2. Accordingly, n denotes the number of drug-pair–cell-line instances and m denotes the feature dimensionality (i.e., m = 1222 in our setting).

#### Joint analysis of class imbalance and overlapping

We define the imbalance ratio (IR) as the proportion of negative to positive samples:

(Equation 4) $$\text{Imbalance}\;\text{Ratio}\;\left(\text{IR}\right)=\frac{\left|N\right|}{\left|P\right|}$$

In our dataset, the imbalance ratio reaches 25.3:1, indicating a pronounced dominance of negatives ({|N|≫|P|}). Table S10 shows that our IR exceeds those in representative prior datasets, e.g., 12.2 (Loewe) and 3.55–3.76 (BLISS/HSA/ZIP) used by Zhang et al.*,*^6^ as well as 15.0 used by Wu et al*.*^8^ Therefore, our setting presents a more severe imbalance, which calls for dedicated imbalanced-learning treatment.

Class imbalance alone cannot capture the full complexity of data distribution.^49^^,^^50^ Local structure, such as overlapping between positive and negative samples, can significantly affect classification. To address both imbalance and overlap jointly, we propose using instance-level hardness and uncertainty measures, which can be efficiently computed and integrated into our model.

#### Instance hardness

Instance hardness reflects which instances are misclassified and provides insight into why they are misclassified and how they contribute to dataset complexity. Instances with low hardness are consistently classified with high confidence, whereas high-hardness instances are frequently misclassified or lie close to the decision boundary. This concept provides insight into dataset complexity and has been shown to be useful for guiding learning and data analysis strategies.^51^

Given the j-th classifier $E_{j}$ (trained on a bootstrap subset $\left(X_{j},Y_{j}\right)$ with parameters $\Theta_{j}$), let p(y∣x) denote the predicted probability that instance x belongs to class y produced by $E_{j}$. For a labeled instance s=⟨x,y⟩, we define its instance hardness under $E_{j}$ as:

(Equation 5) $$IH_{j}\left(x,y\right)=\text{scaler}\left(1-p\left(y|x,E_{j}\left(X_{j},\Theta \right)\right)\right)$$

where x is the feature vector, y is the ground-truth label of x, p(y∣x)∈[0,1] is the predicted probability assigned to the true class by classifier $E_{j}$, and scale(·) is a rescaling function defined below. Larger IH(x,y) indicates greater prediction difficulty.

The average instance hardness across an ensemble of K classifiers is then computed as:

(Equation 6) $$\bar{IH}\left(x,y\right)=\frac{1}{K}\sum_{j=1}^{K}IH_{j}\left(x,y\right)$$

Based on instance hardness, samples can be roughly categorized into three regions: *Stable*, *Critical*, and *Failure*. Stable instances are confidently and consistently predicted, while Failure instances are persistently misclassified and often correspond to noise or outliers. In contrast, Critical instances lie close to the decision boundary, where small variations in the model or training data can change the predicted label. As a result, these instances carry richer information about the boundary location and class separation. Focusing on such Critical instances allows the model to refine the decision boundary more effectively without being distracted by trivially easy samples or noisy outliers. Accordingly, our method primarily emphasizes these Critical instances during training.

To better emphasize subtle differences near the classification boundary (e.g., between predicted probabilities of 0.49 and 0.51), we apply a scaled tanh transformation:

(Equation 7) scaler(τ)=γtanh(δ(τ−0.5))+0.5

where γ and δ control the strength of contrast enhancement. This transformation amplifies distinctions among borderline instances without over-emphasizing trivially easy or noisy samples.

#### Uncertainty measure

Uncertainty is introduced to complement instance hardness by capturing prediction instability across classifiers. Near the decision boundary, small variations in training data or model structure can lead to different classification outcomes, resulting in increased disagreement among classifiers. By quantifying this disagreement, uncertainty helps identify ambiguous boundary instances that are not reliably characterized by average prediction difficulty alone, thereby supporting more reliable decision boundary refinement.^52^ For instance s, we define uncertainty as the variance of IH values across K classifiers:

(Equation 8) $$uncertainty_{s}=\text{Var}\left({\left\{IH_{j}\left(x,y\right)\right\}}_{j=1}^{K}\right)$$

High uncertainty often arises in regions where class distributions overlap, which is common in complex biological datasets such as drug combination data. Notably, instances in the Critical zone tend to exhibit both moderate hardness and high uncertainty, reflecting their proximity to the decision boundary. Rather than emphasizing extreme outliers that are consistently misclassified but exhibit low variability, our model prioritizes these uncertain and borderline instances to better refine decision boundaries and improve generalization.^19^^,^^53^^,^^54^

#### Design of DGBDF

In our model DGBDF, the constructed training set is fed into the first layer $L^{1}$. Each layer contains 4 customized balanced ensemble classifiers. Each balanced ensemble classifier fits by using 5-fold cross-validation within the training set, similar to the original DF.^15^ Each classifier $E_{i}^{1},1\leq i\leq 4$ then outputs a prediction $p\left(y|x,E_{i}^{1}\right)$ for each instance s. Except for the first layer, the predictions of instance s by the previous layer’s classifiers are combined into an augmented vector. These augmented vectors have two functions: (1) Through these augmented vectors, the Balancing Module based on instance hardness and uncertainty measure can resample the initial training samples for the next layer of the DGBDF; (2) Similar to the original DF, the augmented vector of each instance s is concatenated with the original features x to form new features for fitting the next layer of the DGBDF. Subsequent layers grow in the same manner. The growth of DGBDF automatically stops when the performance on the training set no longer improves, and a cascade weighted soft voting method is applied across all layers based on the performance on the training set. The detailed implementation of the DGBDF algorithm is described in Algorithms 1 and 2.

Algorithm 1 **Generation of the l-th layer of DGBDF**

**Require:** Training set D; sampled training set $D_{s}$; number of ensemble classifiers per layer *n*

**Ensure:** Layer L^*(l)*^; augmented features $A^{\left(l\right)}$; instance hardness sets ${IH}^{\left(l\right)}$

1: **function** GENERATELAYER(D, $D_{s}$, n, l)

2:  $L^{\left(l\right)}\leftarrow \emptyset$

3:  $A^{\left(l\right)}\leftarrow \emptyset$

4:  ${IH}^{\left(l\right)}\leftarrow \emptyset$

5:  **for** *i = 1* to *n* **do**

6:  Split *D* into 5-fold stratified CV folds: *{(T*_*j*_*, V*_*j*_*)}*^*5*^_*j=1*_

7:  $T_{j}^{\prime }\leftarrow T_{j}⋂D_{s}$    ▷ retain sampled instances in the training fold

8:  $L_{i}\leftarrow \emptyset$

9:  $A_{i}\leftarrow \emptyset$

10:  ${IH}_{i}\leftarrow \emptyset$

11:  **for** *j = 1* to *5* **do**

12:  Train $E_{i,j}$ by Algorithm $4\left(T_{j}^{\prime },T_{j},20\right)$

13:  Obtain predictions ${\hat{y}}_{i,j}$ for instances in *V*_*j*_

14:  Compute ${IH}_{i,j}$ for instances in *V*_*j*_ by Equation 3

15:  $L_{i}\leftarrow L_{i}\;⋃\left\{E_{i,j}\right\}$

16:  $A_{i}\leftarrow A_{i}\;⋃\left\{{\hat{y}}_{i,j}\right\}$

17:  ${IH}_{i}\leftarrow {IH}_{i}⋃\left\{{IH}_{i,j}\right\}$

18:  **end for**

19:  $L^{\left(l\right)}\leftarrow L^{\left(l\right)}\;⋃\left\{L_{i}\right\}$

20:  $A^{\left(l\right)}\leftarrow A^{\left(l\right)}⋃\left\{A_{i}\right\}$

21:  ${IH}^{\left(l\right)}\leftarrow {IH}^{\left(l\right)}⋃\left\{{IH}_{i}\right\}$

22:  **end for**

23:  **return** $L^{\left(l\right)}$*,* $A^{\left(l\right)}$*,* ${IH}^{\left(l\right)}$

24: **end function**

Algorithm 2 **DGBDF**

**Require:** training set $D^{\prime }$, max depth *d*, threshold ε

**Ensure:** final cascade model F

1: F←∅,H←∅,l←1

2: **while** l≤d **do**   ▷ d is the max depth of the cascade

3:  **if** l=1 **then**

4:  *(*$L^{\left(l\right)}$*,* $A^{\left(l\right)}$*,* $H^{\left(l\right)}$*) ←* 1*(*$D^{\prime }$*,* $D^{\prime }$*, 4)*

5:  **else**

6:  *(*$L^{\left(l\right)}$*,* $A^{\left(l\right)}$*,* $H^{\left(l\right)}$*) ←* 1*(*$D_{s}^{\prime }$*,* $D_{s}^{\prime }$*, 4)*

7:  **end if**

8:  $F\leftarrow F⋃\left\{L^{\left(l\right)}\right\};\;H\leftarrow H⋃\left\{H^{\left(l\right)}\right\}$

9:  Compute $M^{\left(l\right)}$ by Equation 13 (equal layer weights)

10:  **if** l≠1 *and* $M^{\left(l\right)}-M^{\left(l-1\right)}<\varepsilon$ **then**

11:  **Break**

12:  **end if**

13:  $D^{\prime }$ *← concatenate(X,* $A^{\left(l\right)}$*, Y)*

14:  *D′*_*s*_ ← Algorithm 3($D^{\prime }$*,* H*, nl, 5)*

15:  l←l+1

16: **end while**

17: Compute layer weights by Equations 16 and 12

18: **return** $F\left(x\right)=\frac{1}{l}\sum_{j=1}^{l}w^{j}\cdot L^{\left(j\right)}\left(x\right)$

#### Balancing module

The procedure for the Balancing Module is illustrated in Algorithm 3. Its core idea is to iteratively adjust the training data based on the current model’s predictions to better fit the task. It integrates instance hardness and uncertainty to guide sampling.

Algorithm 3 **Balancing Module**

**Require:** Training set D; IH values IH for all instances; number of classifiers K; number of bins b

**Ensure:** Sampled training set $D_{s}$

1: **function** BALANCINGMODULE(D, IH, K, b)

2:  P ← {positive instances in D}

3:  N ← {negative instances in D}

4: ${\;N}_{s}\leftarrow \emptyset$

5:  Compute instance-level mean hardness $\bar{I}\bar{H}$ for N by Equation 4

6:  Compute per-bin capacity c by Equation 8

7:  Compute percentiles $p_{0},p_{1},\ldots ,p_{\beta }$ of $\bar{I}\bar{H}$ **w.r.t.** *c*

8:  Partition N into b bins ${\left\{B_{ᵢ}\right\}}_{i=1^{b}}$ by Equation 9 using $p_{0},p_{1},\ldots ,p_{\beta }$

9:  **for** *i* = 1 to *b* **do**

10:  Compute uncertainty U for instances in $B_{ᵢ}$ by Equation 7

11:  Compute sampling probability *p_f* for each instance in $B_{ᵢ}$ by Equation 10

12:  Sample |P|/b instances $B_{ᵢ}^{\prime }$from *Bᵢ* **w.r.t.** *p*_*f*_

13:  $N_{s}\leftarrow N_{s}⋃B_{ᵢ}^{\prime }$

14:  **end for**

15: ${\;D}_{s}\leftarrow P⋃N_{s}$

16:  **return** $D_{s}$

17: **end function**

To balance attention across instances of varying hardness, we bin negative samples into b bins (Bin-1 to Bin-b) based on their hardness percentiles. Initially, each bin holds $c_{i}=|N|/b$ instances. Over iterations, bin capacities evolve (Figure S3): trivial bins grow while harder bins shrink, bounded below by |P|/b. The bin capacity is calculated via Equation 9, where η is a decay parameter and t is the iteration number. Percentiles $p_{0},p_{1},\ldots ,p_{b}$ are derived from hardness values $\bar{IH}$, defining bins as per Equation 10. Positive samples are placed into Bin-minority.

(Equation 9) $$c_{i}=\frac{\exp\left(-\eta \cdot i\cdot t\right)}{\sum_{j=1}^{b}\exp\left(-\eta \cdot j\cdot t\right)}\cdot \left|P\right|$$

(Equation 10) $$\text{Bin}-i=\left\{\left\langle x,y\right\rangle \;|\;p_{i-1}\leq \bar{IH}\left(x,y\right)<p_{i}\right\},1\leq i\leq b$$

We then use the predictions $\left\{p\left(y\;|\;x,E_{k}\right),1\leq j\leq K\right\}$ of the K sub-classifiers(which could be decision trees or balanced ensemble classifiers) that have previously been constructed to calculate the K instance hardness values {$IH_{j}\left(x,y\right),1\leq j\leq K$} for a single instance s=⟨x,y⟩. The variance of these $IH_{j}\left(x,y\right)$ values is calculated as $uncertainty_{s}$ of the instance, and $uncertainty_{s}$ is used to assign a sampling probability $probability_{s}$ to each instance, as shown in Equation 11, where |B| represents the capacity of the bin to which s belongs. Since subsequent sampling is performed within each bin, normalization is done within each bin. We then sample the same number of instances, |P|/ b, from each of the bins Bin-1, Bin-2, …, Bin-b without replacement. The sampled subset of negative instances is then combined with the positive set from Bin-Minority to form the training set for the next iteration.

Therefore, as the number of iterations increases, more attention is paid to hard-to-classify instances. Note that we do not immediately focus excessively on hard-to-classify instances. The key issue is that misclassification could be caused by noisy or outlier instances, and overemphasizing such instances might lead to erroneous reinforcement. Hence, we should gradually pay more attention to hard-to-classify instances^53^ We utilize all positive samples in each sampling because, compared to negative instances, the number of positive samples is minimal. Initially, the model learns the skeleton of the data, and as it grows, it increasingly focuses on the hard-to-classify instances within the negative class, simultaneously ensuring sufficient attention to instances in the critical zone.

(Equation 11) $$probability_{s}=\frac{uncertainty_{s}}{\sum_{j=1}^{\left|B\right|}uncertainty_{j}}$$

#### Dual granular balancing

Dual Granular Balancing is a core mechanism of DGBDF designed to handle class imbalance throughout the entire learning process, rather than at a single fixed stage. We use the term *dual* to denote two complementary balancing levels, and *granularity* to denote the scale at which rebalancing is applied, spanning from local tree construction to global layer-wise learning. In DGBDF, imbalance can arise both during the construction of individual ensemble classifiers and during the progressive stacking of layers. Accordingly, balancing is performed at two complementary granularities. Inter-tree balancing regulates the sample distribution during the growth of decision trees within each ensemble classifier, while inter-layer balancing adjusts the training samples passed to subsequent layers based on the learning outcomes of earlier layers. By operating at both granularities, the model incrementally corrects imbalance effects from local tree construction to global layer-wise representation learning.

##### Inter-tree balancing

Within each layer of the Deep Forest, there are four balanced ensemble classifiers (BECs). As illustrated in Figure 1, when growing a new decision tree (except the first one) within a BEC, we perform a four-step resampling procedure: (1) compute the instance hardness $IH_{j}\left(x,y\right)$ for each training sample based on the current ensemble state (Equation 6); (2) partition the majority-class samples into b hardness bins (Equation 10) while keeping all minority samples; (3) probabilistically undersample each majority bin according to its sampling probability (Equation 11) to form an updated, more balanced training set; and (4) train the next tree j+1 on this updated set. This resampling–training cycle repeats until the predefined number of trees is reached, resulting in a balanced forest for the BEC. The core idea is to mitigate the adverse effects of class imbalance throughout the training process of each BEC. In this way, each forest module can independently address data imbalance, thereby improving overall robustness. The algorithmic implementation of the BEC is provided in Algorithm 4.

Algorithm 4 **Balanced Ensemble Classifier**

**Require:** Training set T; initial sampled training set $T_{s}$; number of trees n_estimators_

**Ensure:** Balanced ensemble classifier E

1:  **function** BALANCEDENSEMBLE(T, $T_{s}$, n_estimators_)

2:  E←∅

3:  IH←∅

4:  **for** i = 1 to n_estimators_ **do**

5:  Train decision tree $t_{i}$ on $T_{s}$

6:  Compute ${IH}_{i}$ for instances in T by Equation 3

7:  $IH\leftarrow IH\;⋃\;\left\{IH_{i}\right\}$

8:  $T_{s}$ ← **Algorithm 3**(T, IH, i, 5)

9:  $E\leftarrow E\;⋃\;\left\{t_{i}\right\}$

10:  **end for**

11:  **return** E

12: **end function**

##### Inter-layer balancing

DGBDF further performs layer-wise resampling to prevent class-imbalance effects from propagating through the cascade. Using the Balancing Module, We then update the training set for the next layer by resampling positive and negative instances according to these learned instance hardness and uncertainty scores, so that subsequent layers focus more on hard or uncertain samples while maintaining a more balanced class composition. In this way, the inter-layer balancing mechanism transfers the learning signals from earlier layers to later ones and progressively refines the training distribution at the layer level.

The combination of inter-tree and inter-layer balancing is crucial, ensuring a smooth and progressive balancing process. This dual approach ensures that the DGBDF model can effectively handle the imbalance characteristics of drug combination data, thereby improving the performance of predicting synergistic drug pairs.

#### Cascade weighted soft voting

To ensure robust decision-making across the cascade, predictions from different layers are not treated equally but are aggregated based on their relative contribution to overall performance. To decide whether to grow a new layer in the cascade, we compare the model’s current performance with and without the new layer. Unlike the original DF, our framework introduces two key changes.

• Due to severe class imbalance, we adopt f1-macro instead of accuracy as the performance metric.
• As each layer focuses on different samples via the Balancing Module, predictions should be integrated across layers rather than relying on a single one.

Given the severe class imbalance in drug combination data, evaluation metrics should reflect balanced performance across all classes rather than being dominated by the majority class. Unlike accuracy, f1-macro assigns equal importance to each class by averaging class-wise F1 scores, thereby providing a more balanced assessment. We use f1-macro (Equation 16) to evaluate cascade growth. A new layer is added only if the improvement satisfies $M^{l}-M^{l-1}\geq \epsilon$, where $M^{l}$ and $M^{l-1}$ represent model performance with and without the additional layer, and ϵ is a small positive threshold to avoid negligible gains or degradation.

During training, all layers contribute equally when computing $M^{l}$. During testing, predictions from each layer $L^{j}\left(x\right)$ are aggregated via weighted soft voting. Layer weights $W^{l}$ are computed based on their training performance, as shown in (Equation 12), (Equation 13):

(Equation 12) $$W^{l}=\frac{\ln\left(\frac{M^{l}}{1-M^{l}}\right)}{\sum_{j=1}^{\left|L\right|}\ln\left(\frac{M^{j}}{1-M^{j}}\right)}$$

(Equation 13) $$\hat{y}\left(x\right)=\sum_{j=1}^{M}W^{j}\cdot L^{j}\left(x\right)$$

#### Computational complexity

We derive the complexity of DGBDF by decomposing its training procedure into cascade-level construction and tree-level learning under the dual-granularity balancing scheme.

##### Training complexity

DGBDF follows a cascade training paradigm with *L*_*c*_ layers. At each cascade layer, the model consists of *M* forest modules, and each forest contains *T* decision trees. Due to dual-granularity balancing, each tree is trained on an effective training set of size *N′* after under-sampling or reweighting, rather than the full set *N* = *N*^*+*^ + *N*^*−*^. Training a single decision tree on *N′* samples with *d* features has dominant cost O(*N′ d h*), where *h* is the average tree depth, typically *h* = O(log *N*). Therefore, the cost of training one forest is O(*T* · *N′ d h*), and the cost of training all *M* forests within one cascade layer is O(*M* · *T* · *N′ d h*). Aggregating across *L*_*c*_ cascade layers, the overall training complexity scales as O(*L*_*c*_ · *M* · *T* · *N′ d h*). In practice, *L*_*c*_ and *M* are small constants, typically less than 10, and *N′* ≪ *N* in highly imbalanced settings; thus, the training complexity can be well approximated by O(*T N′ d h*) up to a constant factor, which is comparable to other imbalance-aware ensemble methods.

##### Inference complexity

At test time, predicting one sample requires traversing each trained tree with O(*h*) cost per tree. Consequently, inference for one forest costs O(*T h*), for *M* forests within a layer costs O(*M T h*), and for *L*_*c*_ layers costs O(*L*_*c*_ · *M* · *T* · *h*). With *L*_*c*_ and *M* fixed as small constants, the per-sample inference cost reduces to approximately O(*T h*), enabling efficient prediction at scale.

Overall, the complexity comparison in Table S6 reveals clear differences among canonical classifiers, imbalance-aware ensemble methods, and the proposed DGBDF. Canonical tree-based models, including Random Forest, ExtraTrees, Gradient Boosting, and AdaBoost, incur training costs that scale with the full dataset size (*N*^*+*^ + *N*^*−*^), which can become prohibitive under severe class imbalance where *N*^*−*^ ≫ *N*^*+*^. Similarly, *k*-nearest neighbors exhibits linear training cost but suffers from high inference complexity O((*N*^*+*^ + *N*^*−*^) *d*), limiting its scalability at test time. Linear models such as weighted logistic regression and balanced SGD SVM have inference complexity that scales linearly with the feature dimension O(*d*); however, in high-dimensional settings typical of drug combination prediction, this per-sample inference cost can become non-negligible and limit scalability.

In contrast, imbalanced learning ensemble methods, including SelfPacedEnsemble, EasyEnsemble, BalanceCascade, and RUSBoost, reduce training complexity by operating on an under-sampled dataset of size *N′*, yielding training costs of O(*T N′ d h*) while preserving efficient inference O(*T h*). The proposed DGBDF follows a similar favorable scaling behavior: although it introduces additional cascade and ensemble structure, the effective training complexity remains O(*T N′ d h*) due to the use of small cascade depth and a limited number of sub-ensembles. As a result, DGBDF achieves computational efficiency comparable to existing imbalanced learning ensemble methods, while providing enhanced robustness and representational capacity through its cascade architecture.

### Quantification and statistical analysis

To comprehensively evaluate the performance of predicting combination drug classification on such imbalanced datasets, we use sensitivity and specificity to measure the recognition of positive and negative classes, respectively. f1-macro and gmean are also particularly suitable for scenarios involving imbalanced datasets. The f1-macro measures the arithmetic mean of the f1 scores for each class; while gmean measures the geometric mean of sensitivity and specificity.

We also compare the area under the receiver-operating characteristic curve (AUROC) and the area under the precision-recall curve (AUPR). AUROC reflects the ability to distinguish between positive and negative classes across different thresholds, providing a more robust assessment in the presence of data imbalance, while AUPR evaluates the area under the precision-recall curve, focusing on the model’s ability to recognize positive classes. The formulas for these metrics are shown below:

(Equation 14) $$\text{Sensitivity}=\frac{\text{TP}}{\text{TP}+\text{FN}}$$

(Equation 15) $$\text{Specificity}=\frac{\text{TN}}{\text{TN}+\text{FP}}$$

(Equation 16) $$f1-\text{macro}=\frac{1}{2}\sum_{i=1}^{2}2\times \frac{{\text{Precision}}_{i}\times {\text{Recall}}_{i}}{{\text{Precision}}_{i}+{\text{Recall}}_{i}}$$

(Equation 17) $$\text{Gmean}=\sqrt{\text{Sensitivity}\times \text{Specificity}}$$

(Equation 18) $$\text{AUROC}=\int_{0}^{1}\text{TPR}\;d\left(\text{FPR}\right)$$

(Equation 19) $$\text{AUPR}=\int_{0}^{1}\text{Precision}\;d\left(\text{Recall}\right)$$

All statistical analyses were carried out using Python (version 3.9) and relevant libraries, including SciPy(version 1.13.1), NumPy(version 1.24.3), and Pandas(version 2.2.2). For statistical significance, we applied a Friedman test; details of this analysis are provided in the Supplemental Information (Statistical Comparison and Significance Analysis). The number of statistical evaluations for each analysis corresponds to the 5 × 5 repeated 5-fold cross-validation protocol (*N* = 25 blocks). Each block represents one independent train–test split used to evaluate the model. To quantify practical improvements of DGBDF over each baseline beyond rank-based comparison, we computed paired block-wise differences in G-mean and summarized directional consistency using win/tie/loss counts across the 25 blocks. As measures of central tendency, we reported both the mean and median of the paired differences. To quantify uncertainty and precision, we computed bootstrap 95% confidence intervals for the mean and median paired differences by resampling the 25 block-wise differences with replacement (percentile bootstrap). Confidence intervals that exclude zero were used as evidence of consistently positive improvements across repeated cross-validation splits.

For each baseline b, we computed the paired improvement on every block as:

(Equation 20) $$\Delta_{b,i}=G_{\text{ours},i}-G_{b,i},i=1,\ldots ,N$$

Where $G_{b,i}$ denote the G-mean achieved by method m on block i. We also report the mean and median improvements:

(Equation 21) $${\bar{\Delta }}_{b}=\frac{1}{N}\sum_{i=1}^{N}\Delta_{b,i},\Delta_{b}=\text{median}\left(\Delta_{b,1},\ldots ,\Delta_{b,N}\right)\text{.}$$

The percentile bootstrap CIs are given by:

(Equation 22) $${\text{CI}}_{0.95}\left({\bar{\Delta }}_{b}\right)=\left[Q_{0.025}\left({\left\{{\bar{\Delta }}_{b}^{*\left(j\right)}\right\}}_{j=1}^{J}\right),Q_{0.975}\left({\left\{{\bar{\Delta }}_{b}^{*\left(j\right)}\right\}}_{j=1}^{J}\right)\right]\text{,}$$

(Equation 23) $$CI_{0.95}\left({\tilde{\Delta }}_{b}\right)=\left[Q_{0.025}\left({\left\{{\tilde{\Delta }}_{b}^{*\left(j\right)}\right\}}_{j=1}^{J}\right),Q_{0.975}\left({\left\{{\tilde{\Delta }}_{b}^{*\left(j\right)}\right\}}_{j=1}^{J}\right)\right]$$

Where ${\bar{\Delta }}_{b^{*\left(j\right)}}$ and ${\tilde{\Delta }}_{b^{*\left(j\right)}}$ denote the mean and median of the j-th bootstrap resample with the replacement times J = 2000, respectively. And $Q_{q}\left(\cdot \right)$ denotes the q-th empirical quantile.

### Additional resources

This study did not generate additional data.
