# Supplementary material for: A dual granular balanced deep forest model for effective drug combination prediction
Source: iScience. 2026 Jun 3;29(6):116014. doi: 10.1016/j.isci.2026.116014 (PMC13255053; doi:10.1016/j.isci.2026.116014)
Supplement: Document S1. Figures S1–S3 and Tables S1–S10 [file mmc1.pdf]

iScience, Volume *29*

Supplemental information

A dual granular balanced deep forest model for effective drug combination prediction

Zhirui Gong, Ruijiang Li, Kunhong Liu, Yong Xu, Xiaocheng Bo, and Song He

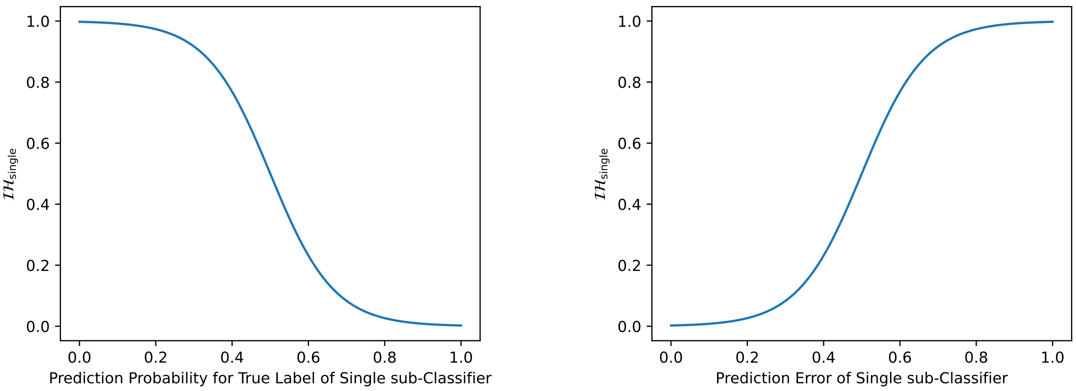

(a) (b)

Figure S1. Relationship between instance hardness and classifier prediction behavior. (a) Visualization of the IH function varying with the instance's prediction probability for the correct label. (b) Visualization of the IH function varying with the instance's prediction error.

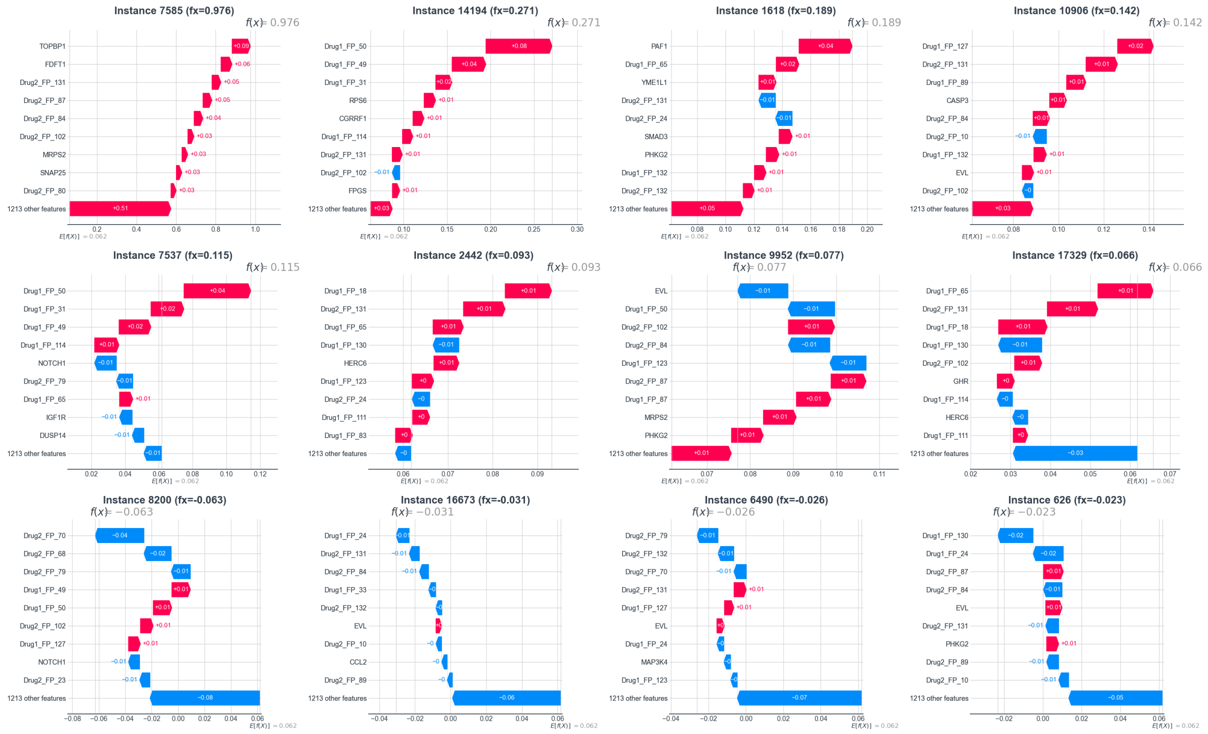

Figure S2. Waterfall plots for 8 randomly chosen positive samples and 4 randomly chosen negative samples, illustrating how different features contribute positively (red) or negatively (blue) to drive the model prediction from the expected value to the final output.

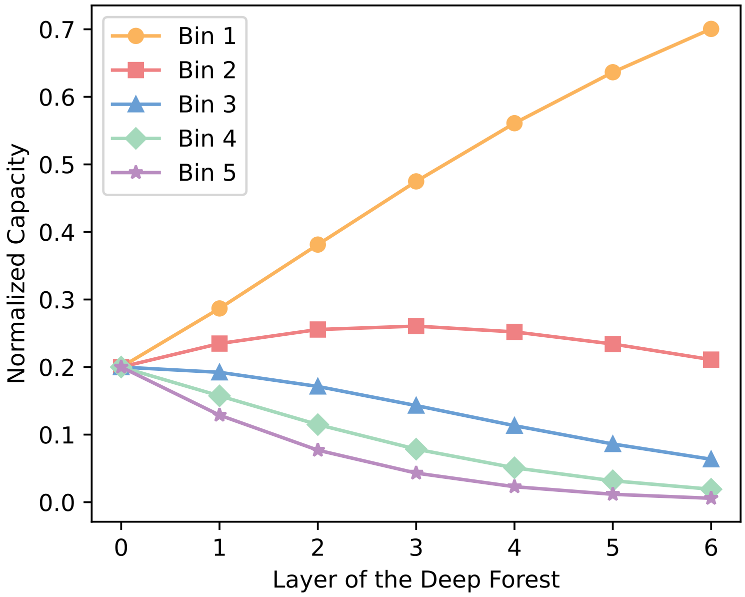

Figure S3: Visualization of Per Negative Bin's Capacity. Bins 1-5 represent the bins from less challenging to more challenging, respectively. The capacity of the less challenging bins gradually increases with the iteration, while the capacity of the more challenging bins decreases.

TABLE S3: Predicted synergistic combinations with AZD7762 in BxPC-3 cell line

**Fixed_Drug**	**Partner_Drug**	**Cell_Line**	**Synergy_Prob**
AZD7762	Camptothecin	BxPC-3	0.73
AZD7762	Navitoclax	BxPC-3	0.68
AZD7762	Sapitinib	BxPC-3	0.59
AZD7762	Afatinib	BxPC-3	0.56
AZD7762	Uprosertib	BxPC-3	0.54
AZD7762	AZD6482	BxPC-3	0.52
AZD7762	Alpelisib	BxPC-3	0.51
AZD7762	ZM447439	BxPC-3	0.49
AZD7762	NU7441	BxPC-3	0.49
AZD7762	Vinorelbine	BxPC-3	0.48
AZD7762	Entinostat	BxPC-3	0.46
AZD7762	Bortezomib	BxPC-3	0.46
AZD7762	JQ1	BxPC-3	0.45
AZD7762	KU-55933	BxPC-3	0.44
AZD7762	Doramapimod	BxPC-3	0.44
AZD7762	GSK269962A	BxPC-3	0.44
AZD7762	PDI73074	BxPC-3	0.43
AZD7762	RO-3306	BxPC-3	0.40
AZD7762	AZD4547	BxPC-3	0.39

Table S1: Summary of Compared Algorithms

Algorithm	Description
Self-Paced Ensemble (SPE) [1]	An ensemble under-sampling method that coordinates sampling with instance hardness via self-paced learning during base classifier training, designed for highly imbalanced datasets.
Easy Ensemble (EE) [2]	Trains multiple base classifiers on different random under-sampled subsets of the majority class and aggregates their predictions.
Balance Cascade (BC) [2]	An extension of EE that guides subsequent under-sampling using previously trained base classifiers to reduce redundant majority instances.
RUSBoost (RUSB) [3]	Combines random under-sampling with boosting by under-sampling the majority class at each boosting iteration.
Weighted Logistic Regression (WLR)	A cost-sensitive logistic regression baseline that assigns higher loss weights to the minority (positive) class (e.g., class weights inversely proportional to class frequencies) to mitigate class imbalance.
Balanced SGD SVM (BSVM)	A cost-sensitive linear support vector machine trained with stochastic gradient descent, where class weights are inversely proportional to class frequencies to address severe class imbalance.
EC-DFR [4]	An enhanced cascade deep forest model proposed for drug combination prediction.
DTF [5]	Learns latent representations of drugs and cell lines via tensor factorization and predicts drug synergy using a neural network.
ProDeepSyn [6]	Integrates PPI networks with multi-omics data and learns low-dimensional embeddings using a GCN to predict anticancer synergistic drug combinations.
ForSyn [7]	A deep forest-based framework with embedded units for imbalance handling and high-dimensional feature processing to predict synergistic drug combinations across cell lines.
ElasticNet [8]	A linear model with elastic-net regularization that predicts the probability of synergistic drug combinations, providing a strong and interpretable baseline with good generalization and flexibility across heterogeneous feature modalities.
HyperGraphSynergy [9]	A knowledge-graph and hypergraph-based deep learning model that learns expressive embeddings via UniGAT, enabling cell line-specific prediction of drug-combination synergy by capturing higher-order biological interactions beyond pairwise modeling.

Table S2: Predicted synergistic drug combinations in HCC1428 cell line

Drug1	Drug2	Cell_Line	Synergy_Prob
Venetoclax	ZM447439	HCC1428	0.82
Navitoclax	Vinorelbine	HCC1428	0.81
Venetoclax	Vinorelbine	HCC1428	0.71
Navitoclax	SB505124	HCC1428	0.68
AZD7762	Irinotecan	HCC1428	0.65
JQ1	Venetoclax	HCC1428	0.60
Camptothecin	Venetoclax	HCC1428	0.57
Erlotinib	Venetoclax	HCC1428	0.56
Dactolisib	Venetoclax	HCC1428	0.55
Doramapimod	Venetoclax	HCC1428	0.53
Sorafenib	Venetoclax	HCC1428	0.52
AZD6482	Venetoclax	HCC1428	0.52
Navitoclax	Ruxolitinib	HCC1428	0.51
GSK269962A	Venetoclax	HCC1428	0.50
Entinostat	Irinotecan	HCC1428	0.50
LGK974	Venetoclax	HCC1428	0.49
Nilotinib	Venetoclax	HCC1428	0.49
Galunisertib	Venetoclax	HCC1428	0.48
Ruxolitinib	Venetoclax	HCC1428	0.48
SB505124	Venetoclax	HCC1428	0.48
Afatinib	Venetoclax	HCC1428	0.48
Entinostat	SCH772984	HCC1428	0.48
Alpelisib	Venetoclax	HCC1428	0.47
Dasatinib	Venetoclax	HCC1428	0.47
Paclitaxel	Venetoclax	HCC1428	0.46
NU7441	Venetoclax	HCC1428	0.46
PF-4708671	Olaparib	HCC1428	0.45
BMS-754807	Olaparib	HCC1428	0.45
Uprosertib	Venetoclax	HCC1428	0.44

TABLE S4: Average ranking of Friedman test based on G-mean.

Model	AvgRank	FinalRank
DGBDF (ours)	**1.0**	**1**
SelfPacedEnsemble	2.0	2
WeightedLogisticRegression	3.0	3
RUSBoost	4.5	4
BalanceCascade	4.5	5
EasyEnsemble	6.0	6
HyperGraphSynergy	7.0	7
PRODeepSyn	8.0	8
BalancedSGDSVM	9.0	9
DTF	10.0	10
ElasticNet	11.5	11
ForSyn	11.5	12
ExtraTrees	13.0	13
RandomForest	14.0	14
GradientBoosting	15.0	15
AdaptiveBoosting	16.0	16
5-NearestNeighbors	17.0	17
MultiLayerPerceptron	18.0	18
EC-DFR	19.0	19

TABLE S8: Summary of Hyperparameter Settings for Imbalanced Learning Algorithms

Model	Hyperparameter	Tuned values
Self-Paced Ensemble	Number of base estimators	30, 50, 100, 200, 500
Easy Ensemble	Type of base estimator	GDBT, Random Forest, Extra Trees, etc.
	Number of base estimators	2, 3, . . . , 10
Balance Cascade	Number of forests	30, 50, 100, 200, 500
RUSBoost	Number of neighbors (k)	3, 5, 7, 9, 11
Weighted Logistic Regression	Max Iteration	2000, 3000, 5000
	Solver	liblinear
	Class Weight	balanced
Balanced SGD SVM	Loss	hinge
	Class Weight	balanced
	Alpha	1e-4
	Max Iteration	2000, 3000, 5000
	Tolerance	1e-3

Table S5: Block-wise improvements of DGBDF over each baseline on G-mean across 25 blocks (5×5 repeated 5-fold CV). We report $\Delta = \mathrm{Gmean}_{\text{ours}} - \mathrm{Gmean}_{\text{baseline}}$, win/tie/loss counts, and bootstrap 95% confidence intervals (CI) for the mean and median improvements.

**Baseline**	**win/tie/loss**	**mean Δ [95% CI]**	**median Δ [95% CI]**	**CI excludes 0**
EC-DFR	25/0/0	0.810 [0.806250, 0.813804]	0.810 [0.803206, 0.816794]	Yes
MultiLayerPerceptron	25/0/0	0.750 [0.746643, 0.753310]	0.750 [0.744090, 0.755910]	Yes
5-NearestNeighbors	25/0/0	0.660 [0.658456, 0.661544]	0.660 [0.657283, 0.662717]	Yes
AdaptiveBoosting	25/0/0	0.640 [0.638456, 0.641544]	0.640 [0.637283, 0.642717]	Yes
GradientBoosting	25/0/0	0.620 [0.619228, 0.620772]	0.620 [0.618641, 0.621359]	Yes
RandomForest	25/0/0	0.570 [0.563150, 0.576946]	0.570 [0.557771, 0.582229]	Yes
ExtraTrees	25/0/0	0.460 [0.455755, 0.464185]	0.460 [0.452527, 0.467473]	Yes
ElasticNet	25/0/0	0.440 [0.435435, 0.444631]	0.440 [0.431848, 0.448152]	Yes
ForSyn	25/0/0	0.430 [0.426141, 0.433913]	0.430 [0.423206, 0.436794]	Yes
DTF	25/0/0	0.400 [0.398810, 0.401158]	0.400 [0.397962, 0.402038]	Yes
BalancedSGDSVM	25/0/0	0.297 [0.294717, 0.299315]	0.297 [0.292924, 0.301076]	Yes
PRODeepSyn	25/0/0	0.280 [0.276913, 0.283087]	0.280 [0.274565, 0.285435]	Yes
HyperGraphSynergy	25/0/0	0.170 [0.165755, 0.174245]	0.170 [0.162527, 0.177473]	Yes
EasyEnsemble	25/0/0	0.100 [0.097685, 0.102250]	0.100 [0.095924, 0.104076]	Yes
BalanceCascade	25/0/0	0.080 [0.079620, 0.080386]	0.080 [0.079321, 0.080679]	Yes
RUSBoost	25/0/0	0.080 [0.079239, 0.080772]	0.080 [0.078641, 0.081359]	Yes
WeightedLogisticRegression	25/0/0	0.070 [0.067749, 0.072315]	0.070 [0.065924, 0.074076]	Yes
SelfPacedEnsemble	25/0/0	0.040 [0.038098, 0.041875]	0.040 [0.036603, 0.043397]	Yes

TABLE S9: Summary of Hyperparameter Settings for Existing Drug Combination Prediction Algorithms

**Model**	**Hyperparameter**	**Tuned values**
EC-DFR / ForSyn	Number of base estimators	30, 50, 100, 200, 500
	Number of forests	2, 4, 6
	Error threshold (percentile rank)	10%, 20%, . . . , 90%
	Forest type	GDBT, Random Forest, Extra Trees, etc.
DTF	Hidden units	[1024, 1024, 512]; [2048, 2048, 1024]; [2048, 1024, 512]; [2048, 2048, 2048]
	Learning rate	1e-2, 1e-3, 1e-4, 1e-5
	Dropout	No dropout; input: 0.1, 0.2; hidden: 0.1, 0.2, 0.3, 0.4, 0.5
PRODeepSyn	Gene's embedding space	32, 64, 128, 256, 512
	Cell line's embedding space	128, 320, 384, 448, 512
	Error threshold (percentile rank)	10%, 20%, . . . , 90%
ElasticNet	epoch	50, 100, 200, 500
	Learning rates	1e-2, 1e-3, 1e-4, 1e-5
	Loss	LSE, BCE
	Activation	sigmoid, relu
HyperGraphSynergy	epoch	50, 100, 200, 500
	Learning rates	1e-2, 1e-3, 1e-4, 1e-5

Table S6: Computational complexity comparison. Here, N^+ and N^- denote the numbers of positive and negative training samples, respectively, with $N = N^+ + N^-$. For imbalance-aware methods, N' denotes the effective training set size after under-sampling (typically $N' \approx 2N^+$ or $N' \ll N$). d is the feature dimension, T the number of trees per ensemble, h the average tree depth (typically $h = \mathcal{O}(\log N)$), K the number of nearest neighbors, and L_c the number of cascade layers. Training and inference complexities report the dominant time cost.

**Method**	**Training**	**Inference (per sample)**
Random Forest	$\mathcal{O}(T(N^+ + N^-)d\,h)$	$\mathcal{O}(Th)$
k-Nearest Neighbors	$\mathcal{O}((N^+ + N^-)d)$	$\mathcal{O}((N^+ + N^-)d)$
ExtraTrees	$\mathcal{O}(T(N^+ + N^-)d\,h)$	$\mathcal{O}(Th)$
GradientBoosting	$\mathcal{O}(T(N^+ + N^-)d\,h)$	$\mathcal{O}(Th)$
AdaptiveBoosting	$\mathcal{O}(T(N^+ + N^-)d\,h)$	$\mathcal{O}(Th)$
SelfPacedEnsemble	$\mathcal{O}(TN'd\,h)$	$\mathcal{O}(Th)$
EasyEnsemble	$\mathcal{O}(TN'd\,h)$	$\mathcal{O}(Th)$
BalanceCascade	$\mathcal{O}(TN'd\,h)$	$\mathcal{O}(Th)$
RUSBoost	$\mathcal{O}(TN'd\,h)$	$\mathcal{O}(Th)$
Weighted Logistic Regression	$\mathcal{O}((N^+ + N^-)d)$	$\mathcal{O}(d)$
Balanced SGD SVM	$\mathcal{O}(E(N^+ + N^-)d)$ (SGD epochs E)	$\mathcal{O}(d)$
DGBDF (ours)	$\mathcal{O}(TN'd\,h)$	$\mathcal{O}(Th)$

TABLE S7 Summary of Hyperparameter Settings for Canonical Algorithms

**Model**	**Hyperparameter**	**Tuned values**
Random Forest / Extra Trees	Number of base estimators	30, 50, 100, 200, 500
Adaptive Boosting	Type of base estimator	GDBT, Random Forest, Extra Trees, etc.
	Number of base estimators	2, 3, . . . , 10
Gradient Boosting	Number of base estimators	30, 50, 100, 200, 500
5-Nearest Neighbors	Number of neighbors (k)	3, 5, 7, 9, 11
Multi-Layer Perceptron	Hidden layer sizes	(50,), (100,), (100, 50), (100, 100)
	Activation function	ReLU, tanh, logistic
	Learning rate	0.001, 0.01, 0.1

Table S10: Imbalance Ratio Comparison

Dataset	Samples	Synergy Type	Imbalance Ratio
Jaaks's	57,246	–	25.3
[10]	204,712	Loewe	12.2
[10]	204,712	BLISS	3.59
[10]	204,712	HSA	3.76
[10]	204,712	ZIP	3.55
[7]	3,192	–	15.0

References

[1] Zhining Liu, Wei Cao, Zhifeng Gao, Jiang Bian, Hechang Chen, Yi Chang, and Tie-Yan Liu. Self-paced ensemble for highly imbalanced massive data classification. In *2020 IEEE 36th international conference on data engineering (ICDE)*, pages 841–852. IEEE, 2020.

[2] Xu-Ying Liu, Jianxin Wu, and Zhi-Hua Zhou. Exploratory undersampling for class-imbalance learning. *IEEE Transactions on Systems, Man, and Cybernetics, Part B (Cybernetics)*, 39(2):539–550, 2008.

[3] Chris Seiffert, Taghi M Khoshgoftaar, Jason Van Hulse, and Amri Napolitano. Rusboost: A hybrid approach to alleviating class imbalance. *IEEE transactions on systems, man, and cybernetics-part A: systems and humans*, 40(1):185–197, 2009.

[4] Weiping Lin, Lianlian Wu, Yixin Zhang, Yuqi Wen, Bowei Yan, Chong Dai, Kunhong Liu, Song He, and Xiaochen Bo. An enhanced cascade-based deep forest model for drug combination prediction. *Briefings in Bioinformatics*, 23(2):bbab562, 2022.

[5] Zexuan Sun, Shujun Huang, Peiran Jiang, and Pingzhao Hu. Dtf: deep tensor factorization for predicting anticancer drug synergy. *Bioinformatics*, 36(16):4483–4489, 2020.

[6] Xiaowen Wang, Hongming Zhu, Yizhi Jiang, Yulong Li, Chen Tang, Xiaohan Chen, Yunjie Li, Qi Liu, and Qin Liu. Prodeepsyn: predicting anticancer synergistic drug combinations by embedding cell lines with protein–protein interaction network. *Briefings in bioinformatics*, 23(2):bbab587, 2022.

[7] Lianlian Wu, Jie Gao, Yixin Zhang, Binsheng Sui, Yuqi Wen, Qingqiang Wu, Kunhong Liu, Song He, and Xiaochen Bo. A hybrid deep forest-based method for predicting synergistic drug combinations. *Cell Reports Methods*, 3(2), 2023.

[8] Lea Eckhart, Kerstin Lenhof, Lutz Herrmann, Lisa-Marie Rolli, and Hans-Peter Lenhof. How to predict effective drug combinations–moving beyond synergy scores. *iScience*, 28(6), 2025.

[9] Maryam Mehrabani, Amir Lakizadeh, Alireza Fotuhi Siahpirani, Mahdieh Salimi, Fatemeh Zare-Mirakabad, and Ali Masoudi-Nejad. Synergygraph: predicting cell line specific drug combination synergy scores using knowledge graph representation and hypergraph modeling. *Scientific Reports*, 2025.

[10] Peng Zhang and Shikui Tu. A knowledge graph embedding-based method for predicting the synergistic effects of drug combinations. In *2022 IEEE international conference on bioinformatics and biomedicine (BIBM)*, pages 1974–1981. IEEE, 2022.
